# Editorial: Special Issue “Liquid Crystals 2020”

**DOI:** 10.3390/molecules28083359

**Published:** 2023-04-11

**Authors:** Viorel Cîrcu, Doina Manaila-Maximean, Valery A. Loiko

**Affiliations:** 1 Department of Inorganic and Organic Chemistry, Biochemistry and Catalysis, Faculty of Chemistry, University of Bucharest, Boulevard Regina Elisabeta Nr. 4-12, 030018 Bucharest, Romania; 2 Department of Physics, Universitaty Politehnica of Bucharest, 313 Spl. Independentei, 060042 Bucharest, Romania; 3 B.I. Stepanov Institute of Physics, National Academy of Sciences of Belarus, 68-2 Niezalezhnastsi Avenue, 220072 Minsk, Belarus

This Special Issue, entitled “Liquid Crystals 2020”, is a collection of ten original research papers, including two feature papers, on theoretical and experimental advanced studies of liquid crystal science and technology.

In recent years, studies have focused on the development of new liquid crystals (LCs), LC composites, and their applications. Of primary importance is the understanding of the effects that occur at phase transitions in pure and doped LCs.

Novotna et al. [1], in studying the texture of an LC doped with a cobalt–ferrite magnetic in the SmA phase, below the pure LC Isotropic-SmA transition temperature, found a broad-range coexistence of the isotropic and SmA phases induced by clusters of nanoparticles (NPs), attributed to the observed regular defect system. It was considered that the cluster formation originates in the interaction of NPs with dislocation segments, rather than due to magnetic forces, and they concentrate in the centers of some dislocation loops. The cluster nuclei interact and grow by attracting more NPs until a critical radius is attained when the texture is stabilized. Thus, the inclusions interact at a much larger distance than the NP size. The formation of clusters is also observed at the interruption of the electric field for this liquid crystal with positive dielectric anisotropy.

Additionally, in studying the same topic of phase transitions, Kowaguchi et al. [2] performed temperature and pressure replica-exchange MC simulations of an LC simple model. They reported a second-order isotropic–nematic phase transition and the complete absence of a stable smectic phase for the range of temperatures and pressures investigated, together with first-order direct nematic to the solid phase transition. The results showed how the replica-exchange method enables us to explore a broad range of phase spaces, which effectively samples the equilibrium and provide an efficient way of accessing wider free-energy regions. This approach is also more computationally efficient for non-spherically symmetric molecules when compared to the traditional MC method, the algorithm being useful for the study of complex materials.

In the era of NPs, the study of electro-optical effects in new composite materials based on LC-doped NPs is a necessity [3].

Of particular importance in the study of electro-optical effects in LCs is the Freedericksz transition. In an LC sample under the influence of an electric field, each element of volume is subjected to a dielectric coupling of forces, tending to align the molecules under a certain direction, and an elastic coupling, due to the interaction of the LC with the surfaces that impose the initial orientation; the two force couples are in competition and the change in the orientation of the molecules will occur only when the value of the applied voltage exceeds a certain threshold value. In her work [4], Cirtoaje developed a theoretical model to describe the influence of surface anchoring on the Freedericksz transition threshold and saturation field. The model considers the planar configuration of positive dielectric anisotropy LCs doped with barium titanate ferroelectric NPs. The experiments carried out support the developed model. Freedericksz transition threshold voltages versus anchoring strength energy for different NP-LC interaction energies were obtained, both theoretically and experimentally. By choosing functionalized ferroparticles, the electric Freedericksz transition can be considerably reduced without an increase in NP concentration. The surface treatment of the substrate is also important because an equilibrium between good alignment and rapid reorientation is crucial for high-performance devices.

Knowing the dielectric permittivity is useful in the study of LC electro-optical effects, and also in the design of electrical circuits [5,6]. Manaila-Maximean [7] studied the effective dielectric properties of heterogeneous materials of the type of quasi-spherical particle inclusions in a host medium, using the Maxwell Garnet and Bruggeman theories. The results of the theories are applied to polymer-dispersed liquid crystal (PDLC) films, nanoparticle (NP)-doped LCs, and developed for NP-doped PDLC films. The effective permittivity of the composite was simulated at sufficiently high frequency, where the permittivity is constant, obtaining results on its dependency on the constituents’ permittivity and concentrations. The two models and experimental results were compared and discussed. The method also retains a general character and can be applied to other similar multiphase composites.

Modern life and technology rely on large LC waste amounts that, if improperly treated, might become an environmental problem. Thus, the study of the physico-chemical properties of recycled LC is of utmost importance. In [8], Barrera et al. studied the dielectric properties of recycled nematic liquid crystals, at room temperature, in low- and medium-frequency ranges.

Results show that the dielectric anisotropy of all purified samples presents positive values and decreases after the addition of diamond NPs to the LC mixtures. DC conductivity values were obtained by applying the universal law of dielectric response proposed by Jonscher.

The conductivity of the doped LC mixtures is lower than that of the undoped and non-purified LC. Adding the diamond NP leads to a decrease in ionic conductivity due to the trapping of ionic impurities on their surface. A small amount of DNPs has been shown to reduce the ionic conductivity of the sample by two orders of magnitude compared to the non-purified samples. Consequently, such recycled LC material could be upgraded for further technology uses.

The Lehman effect, consisting of the rotation of a cholesteric droplet under heat gradient, was the starting point of the study by Kiang-ia et al. [9], in which a radial achiral nematic LC droplet was trapped using linearly polarized optical tweezers. During the laser trapping experiment, symmetry breaking in a radial hedgehog defect in the middle of the NLC droplets occurs and the defect is pushed off the center. The droplet, although composed of achiral molecules, was distorted due to the laser polarization direction. When increasing the laser power, a stable trap was observed, followed by a reverse directional rotation in a higher-intensity laser trap. The location of the droplet in the trap is the result of the competition of the gravitational force and the laser radiation pressure. The rotational sense of the droplet depends on the heat gradient direction, which can be determined by the location of the droplet on the laser beam axis with respect to the laser focal spot.

Sarukhanyan et al. [10] studied, both experimentally and theoretically, the spectral characteristics and lasing capabilities of a dye-doped polymer layer (DDPL) embedded in a wedge-shaped cholesteric liquid crystal (CLC) cell in light of the growing interest in the study of optical properties of CLCs with induced defects. The theoretical results reveal that defect modes can be generated both periodically and continuously along specific spectral lines inside the photonic bandgap (PBG). This paper reports, for the first time, a robust spectral behavior of induced defect modes, which is directly related to the geometric-phase character of CLC helices surrounding the isotropic defect layer. This work suggests great potential applications for low-threshold robust lasing, multi-position triggers, filters, etc., by implementing the gradient-pitch CLCs in the wedge-shaped cell to widen its PBG and, consequently, the spectral range of multimode lasing.

Liquid crystals have been extensively exploited in the construction of LC biosensors based on the idea that certain bonding interactions between biomolecules can influence the alignment of LC molecules. In this Special Issue, Abbasi et al. [11] highlighted the use of LCs in biological detection by developing a unique biosensing platform with tremendous potential to be explored further for the manufacture of a point-of-care device for the rapid detection of HIV-1. The researchers described a label-free and efficient method for detecting glycoprotein-120 (gp-120, the HIV-1 envelope glycoprotein, which has an essential function in HIV infection) through an aptamer-based liquid crystals biosensors by using aptamer B40t77 as a molecular probe. The new biosensing platform has a detection limit for gp-120 of 0.2 g/mL and high selectivity, as no response was detected for other proteins, such as bovine serum albumin (BSA), hepatitis A virus capsid protein 1 (Hep A VP1), and immunoglobulin G protein (IgG).

The chemistry of LCs is a fundamental domain in liquid crystals science because new materials must be designed and produced to meet the increasing requirements for their specific focused applications. Two interesting contributions, one by Bruce et al. [12] in the field of ionic liquid crystals (ILCs), and a second by Rieth et al. [13] in the field of discotic liquid crystals (DLCs) based on star-shaped compounds, were included in the present collection. ILCs are now being widely investigated due to their great characteristics that come from the combination of LC and ionic liquid (IL) properties [14,15]. The application of ILCs in solar cells, membranes, battery materials, electrochemical sensors, electroluminescent switches, etc. is now well known. The structural design of ILCs is crucial to the type of mesophases displayed by these compounds and is governed by a variety of parameters, including molecule shape, position, size of ionic groups, intermolecular interactions, and microphase segregation. The mesomorphism of tetracatenar ILCs salts based on a rigid N-phenylpyridinium core, with various counterions, such as triflimide, octyl sulfate, and dodecyl sulfate, is described in [12]. This work perfectly illustrates how the interplay between electrostatic interactions, charge density, and the steric effects of both anions and terminal chains can affect the properties of LCs. For shorter terminal chains, SmA phases were observed, while longer chains gave way to hexagonal columnar phases (Col_h_) with an unexpected long-range mesophase stabilization. Interestingly, the alkyl sulfate anions can also give an intermediate cubic phase. An extensive study regarding the impact of the number of alkyl chains, their position, length, and structure on the thermal behavior of tristriazolotriazines (TTTs) compounds with a threefold alkoxyphenyl substitution is presented in [13]. The experimental results showed that the substitution pattern has a profound effect on the thermal properties of TTTs. Although these compounds display broad and stable thermotropic LC phases, the authors found that the properties of tangential TTTs are superior to those of the related radial TTT isomers.
